# Supplementary material for: How Italy Tweeted about COVID-19: Detecting Reactions to the Pandemic from Social Media
Source: Int J Environ Res Public Health. 2022 Jun 24;19(13):7785. doi: 10.3390/ijerph19137785 (PMC9265594; doi:10.3390/ijerph19137785)
Supplement: Supplementary file 1 [file ijerph-19-07785-s001.zip › ijerph-1752989-supplementary.pdf]

Table S1. Selection of themes for the semantic analysis.

Wang et al. (New York)	Wang et al. (California)	Our study
Protective Measures	Protective Measures	Preventive measures
Pandemic's impact	The pandemic of COVID-19	Virus and pandemic impact
Government and Policy	Government and Policy	Geopolitics and government
Public concern	Public concern	Community
Impact on life	Impact on public resources	Community
Hospital situation	Hospital situation	Medical support
Testing and vaccine	Test results	Medical support
Confirmed cases and deaths	Confirmed cases and deaths	Virus and pandemic impact
Medical support	-	Medical support
Government response	-	Geopolitics and government
School closure	-	Community
-	State orders	Geopolitics and government
The impact of COVID-19	-	Virus and pandemic impact
-	Bill relief	Community
-	Vaccine and Immunization	Medical support
-	Global news	Information seeking
-	Quarantine	Preventive measures

Table S2. Mean daily frequency of tweet posted by week, 25 February – 4 May 2020.

	All	Verified	Non-verified
Week 1: 02/25 - 03/01/2020	21,720	222	21,498
Week 2: 03/02 - 03/08/2020	69,104	1,097	68,007
Week 3: 03/09 - 03/15/2020	131,563	2,030	129,532
Week 4: 03/16 - 03/22/2020	96,124	2,002	94,122
Week 5: 03/23 - 03/29/2020	85,313	2,057	83,256
Week 6: 03/30 - 04/05/2020	69,476	1,834	67,642
Week 7: 04/06 - 04/12/2020	55,671	1,701	53,970
Week 8: 04/13 - 04/19/2020	56,541	1,559	54,982
Week 9: 04/20 - 04/26/2020	56,641	1,548	55,093
Week 10: 04/27 - 05/03/2020	62,743	1,338	61,404

Table S3. Spearman rho correlation between frequency of tweets expressing emotions and frequency of mentions of bigrams related to the different themes identified with the semantic analysis.

**Emotion**	**Community**	**Information seeking**	**Medical support**	**Geopolitics and government**	**Preventive measure**	**Time**	**Virus and pandemic impact**
Anger	-0.632	-0.055	-0.006	-0.104	0.129	0.202	0.558
Anticipation	0.152	0.389	0.140	-0.122	-0.249	0.486	-0.061
Disgust	-0.042	-0.297	-0.309	-0.018	0.176	-0.030	0.055
Fear	-0.249	**-0.650***	-0.109	-0.231	0.413	-0.079	-0.073
Joy	0.460	**0.669***	0.528	0.374	**-0.724***	0.067	-0.012
Sadness	-0.450	-0.584	-0.012	-0.249	0.413	0.140	0.073
Surprise	-0.037	0.425	**0.843***	-0.025	-0.357	0.369	-0.012
Trust	0.455	0.588	0.430	0.515	**-0.879***	-0.1224	0.115

*P-value<0.05

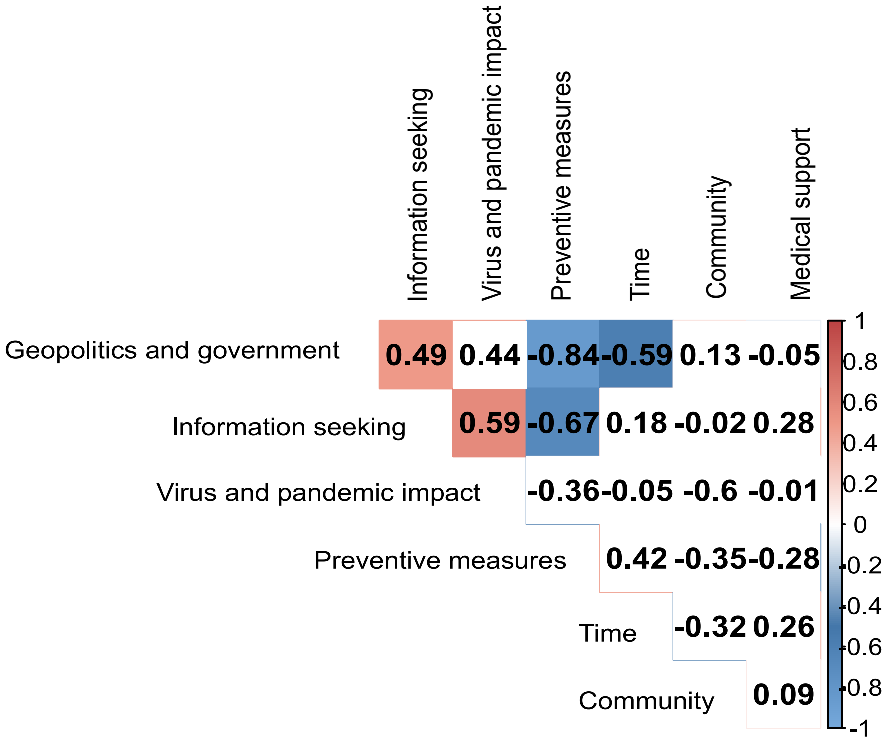

Figure S1. Pairwise correlation between themes identified through the semantic analysis.

The Spearman rho correlation coefficients and statistically significant correlations are highlighted in red (for positive correlations) and blue (for negative correlations).

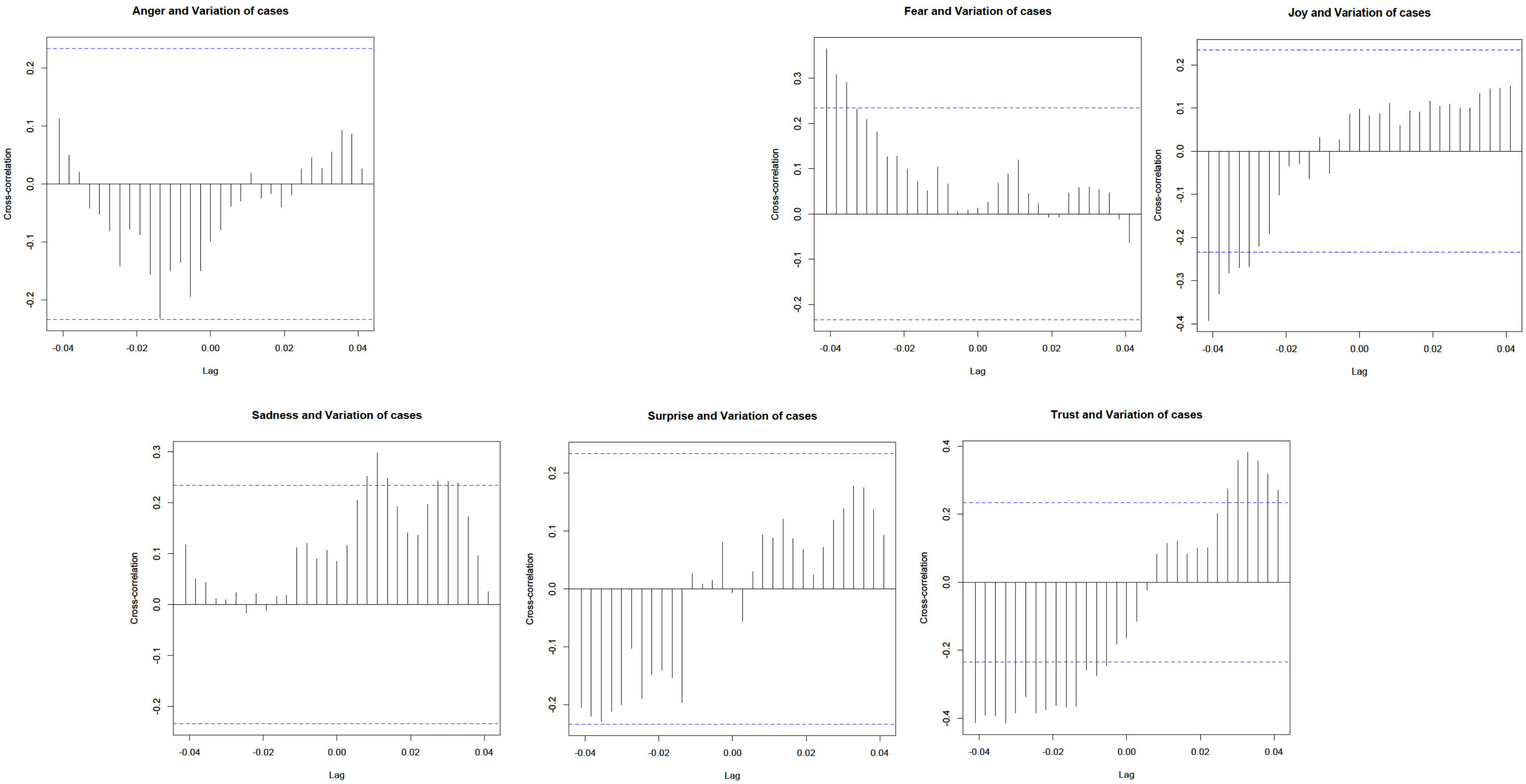

Figure S2. The cross-correlation function between each emotion and variations in newly-confirmed cases.

The horizontal blue lines in each graph represent the estimated 95% confidence interval on the cross-correlation function and thus help to identify significant correlations (those exceeding the interval) as well as various negative and positive time lags.

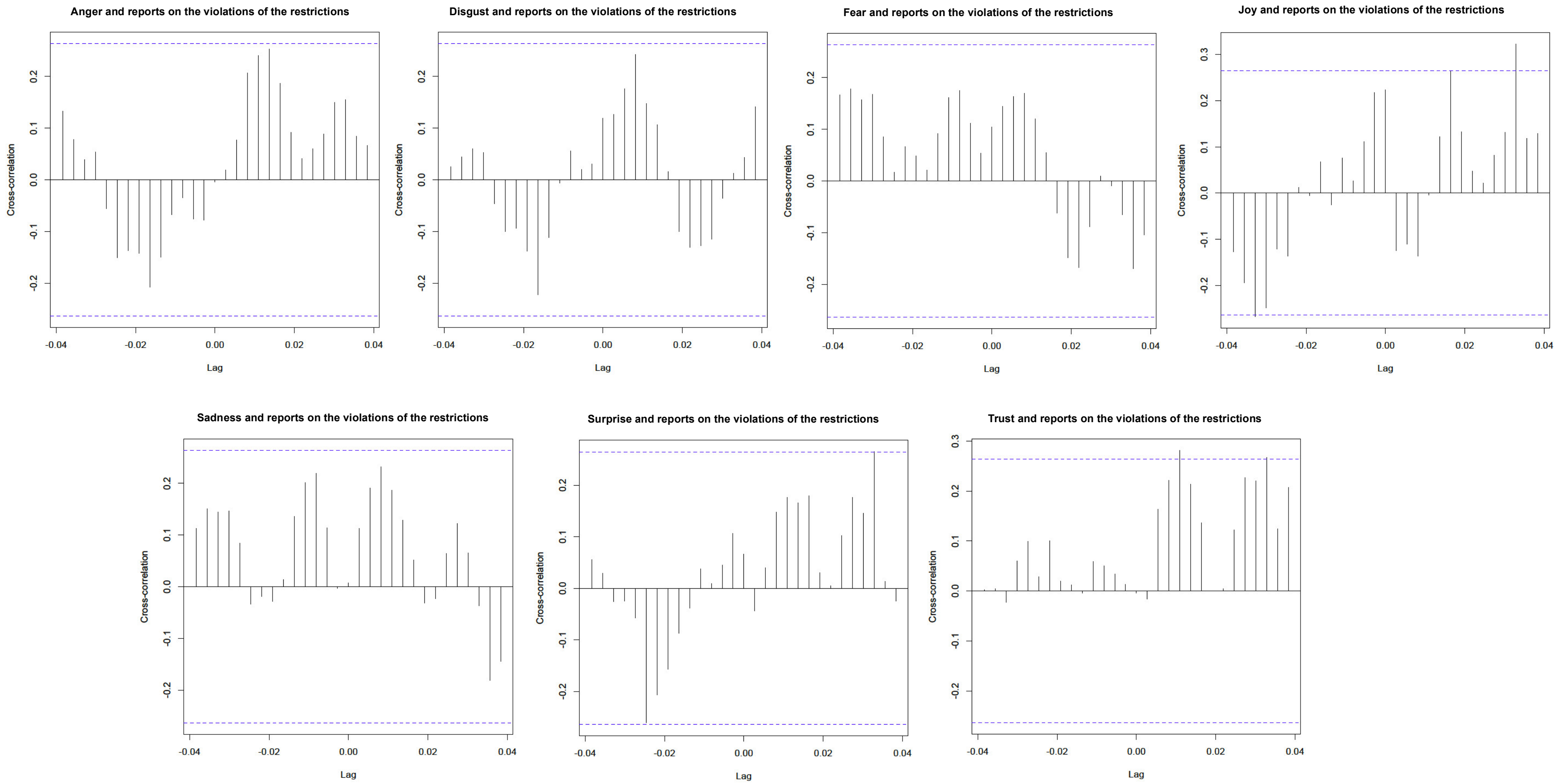

Figure S3. The cross-correlation function between each emotion and daily number of subjects fined for specific violations of restrictions. The horizontal blue lines in each graph represent the estimated 95% confidence interval on the cross-correlation function and thus help to identify significant correlations (those exceeding the interval) as well as various negative and positive time lags.
